# Advancing whose interests? Corporate strategy and risks to food systems transformation and public health nutrition through academic partnerships in Africa

**DOI:** 10.1136/bmjgh-2024-016049

**Published:** 2025-01-19

**Authors:** Safura Abdool Karim, Busiso Moyo, Helen Walls

**Affiliations:** 1 Berman Institute of Bioethics, Johns Hopkins University, Baltimore, Maryland, USA; 2 Centre for the AIDS Programme of Research in South Africa, Doris Duke Medical Research Institute, University of KwaZulu-Natal, Durban, South Africa; 3 Global Health and Development, London School of Hygiene & Tropical Medicine, London, UK; 4 Institute for Social Development, Faculty of Economic and Management Sciences, University of the Western Cape, Cape Town, South Africa

**Keywords:** Nutrition, Accountability, Health policy, Public Health, Decision Making

**SUMMARY BOX**

- Food systems contribute to hunger, malnutrition in all its forms and environmental harm, and, particularly in Africa, are vulnerable to climate and other global and regional shocks, as seen during COVID-19. There is an urgent need to transform food systems through evidence-based interventions. Corporate influence in food systems is a critical barrier to meaningful reform of the food system. Multinational corporations (MNCs) often instrumentalise research and evidence, including by co-opting academics, to influence public health policies and interventions in ways that prioritise profit over public health.
- This study highlights how, despite recognition of the problems of MNC involvement in research, corporate academic partnerships, such as the recent collaboration between Nestlé and the University of Pretoria, continue to allow MNCs to strongly shape research agendas. This is particularly concerning in Africa, where achieving a food system transformation is of critical importance. The study underscores that corporate-funded research risks perpetuating biased evidence and policies that align more with corporate interests than with the needs of public health and food system transformation.
- This study calls for heightened scrutiny, and potentially avoidance, of industry-academic partnerships in public health nutrition and wider food system research. This call has particular significance for African contexts characterised by resource constraints and limited funding. It suggests that public health practitioners and policymakers reassess the role of corporations in food systems research to safeguard the quality of research evidence, unaffected by industry biases. Such scrutiny could inform regulations that minimise corporate influence, thereby supporting the generation of research evidence to support the achievement of transformative change in food systems and public health nutrition so urgently needed.

## Introduction

Food systems globally are contributing to inequitable burdens of hunger and malnutrition. Simultaneously, food systems are contributing to environmental degradation, while also being vulnerable to climate change and other global and regional shocks, as evidenced by supply chain disruptions during the COVID-19 pandemic.^1 2^ Radical change is needed in food systems to address these consequences and vulnerabilities. This is especially so in Africa, where in 2022, 123 million people (12% of sub-Saharan Africa’s population) were projected to face acute food insecurity, with high malnutrition and insufficient food consumption.^3^ Key to transforming food systems are evidenced-based interventions that shift the focus from technical aspects such as improving agricultural productivity or nutrient supplementation to addressing the significant power imbalances that influence global food system governance and outcomes. Understanding and tackling power imbalances in food systems are central to achieving a transformation that addresses the root causes of the multiple burden of malnutrition and related food system outcomes.^4^

Agri-food corporations influence food system decision-making and dominate seemingly inclusive ‘multi-stakeholder’ processes and thus, in particular, continue to unduly shape the institutions, policies and norms that govern our food systems.^5^ From shaping the design of healthy diet initiatives to infiltrating high-level advisory bodies and co-opting academia, corporate influence on food systems is pervasive.^5^ Such power relations shape how food is produced, acquired, prepared and consumed; pointedly, this translation of the market power of large multinational corporations (MNCs) into self-serving political power in which they gain political legitimacy as economic actors in the food chain is the dominant barrier to action to achieve a food systems transformation.^5^^4^

The influence of corporations on public policy, described as corporate political activity, poses a significant challenge to efforts aimed at preventing non-communicable diseases. This is particularly evident in the case of several industry types, including the processed food industry, where MNCs’ covert ‘below-the-line’ activities often result in the adoption of watered-down public health interventions. The significant market power influence wielded by leading MNCs poses a public health issue as it grants them the authority to shape and perpetuate food supply chains and regulatory environments that prioritise the creation and consumption of their often heavily processed products. Thus, the profit interests of MNCs are prioritised over the public’s health.^5^ Such practices have prompted public health practitioners to advocate for the exclusion of corporations producing unhealthy products from the development of public health policies and research. This approach aims to safeguard public health objectives from being undermined by commercial interests, ensuring that policy decisions are genuinely in the best interests of the population’s health.

On 16 November 2023, South Africa’s University of Pretoria (UP), which co-hosts the country’s Centre of Excellence in Food Security, announced what they termed a ‘transformative’ partnership with Nestlé, the world’s largest food company, to advance food and nutrition research in Africa.^6^ This new Nestlé-UP partnership is building on an already well-established tradition.

The Nestlé-UP partnership will see Nestlé actively involved in supporting research conducted by the African Research Universities Alliance (ARUA) Centre of Excellence in Sustainable Food Systems, a consortium of African institutions, through funding research on food and nutrition as well as hosting and mentoring postgraduate students.^6^ Thus, the partnership will enable the corporation to shape research agendas and the direction of future research on food and nutrition in Africa.

The partnership has been framed positively by those involved, but corporate activities should be critically analysed given well-documented histories of often being undertaken at the expense of public health.^7^ Certainly, it is important to consider what role, if any, industry, and in this case the food industry, should have in supporting academic research. Previously, concerns were raised about whether the relationship between Nestlé and ARUA’s prior director constituted a conflict of interest,^8^ and this latest development is evidence of a contentious relationship deepening into a fully fledged collaboration.

The Nestlé-UP partnership is certainly not the first time a corporation, or even Nestlé itself, has funded research activities. In 2021, concerns about a conflict of interest arising from a member of ARUA serving on the board of Nestlé were flagged and the relationship criticised by many academics.^9^ This reflects how business actors have a long history of co-opting researchers and academics to further their interests.^10^ Given the aims and incentives of industry actors, this is to be expected. Industry works towards maximising profit, and when public health goals such as calls for a stronger regulatory environment to curb highly processed food consumption might compromise the industry’s bottom line, corporations will unsurprisingly take steps to protect their business interests and in the process undermine public health. Corporate actors use a ‘playbook’ of strategies to advance their financial interests through achieving more favourable regulatory environments. This includes funding research that downplays the harmful effects of or deflects attention from their products, creating doubt in the science, and co-opting scientists to advocate for corporate interests.^7 11^ These partnerships enable industry to use researchers, including university academics, as part of a political strategy to shape the evidence base and narratives that influence policy decision-making—cloaking themselves in the legitimacy of academia and science while ultimately acting to undermine public health.^12^

On its website, Nestlé itself highlights a range of such collaborations beyond UP,^13^ describing how they ‘work closely with a wide range of academic institutions and public organisations worldwide’. Nestle then highlights its academic partnerships with groups such as the EpiGen Consortium (Singapore, the UK and New Zealand) that addresses infant and young child feeding, ETH Zurich and École Polytechnique Fédérale de Lausanne with whom Nestlé is a founding member of the Future Food Initiative, the European Masters of Food Studies at Wageningen University & Research and the University of Ghana, where Nestlé supports PhD students addressing issues of public health nutrition and highlights how this relationship builds on the company’s ‘strong collaboration’ with sub-Saharan African academic networks.

In the 2010s in South Africa, while the government was seeking to address the use of infant formula through implementing comprehensive regulation of the product, producers of infant formula, including Nestlé, were heavily sponsoring events at universities and health associations, and promoting incorrect information through active targeting of healthcare providers.^14^ Yet again, this Nestlé-UP collaboration—as an institutionally well-embedded activity on the part of a major food industry player, wrapped in the guise of advancing food and nutrition research in Africa—represents a significant use of corporate strategy to further industry interests. Importantly, it also signals Nestlé’s further efforts to expand industry influence in Africa—one of the company’s programme managers has described the collaboration as ‘part of ongoing efforts to strengthen scientific engagement with universities and research institutes in sub-Saharan Africa’. South Africa’s role as a gateway for unhealthy-commodity-producing industries to infiltrate the less-regulated markets of sub-Saharan Africa has been noted previously.^15^

Generally, public health scholars hold the view that the use of evidence in policymaking and governance is inherently positive (the so-called evidence-based or more realistically, evidence-informed policy discourse).^16^ Such views often assume that this involves the generation of neutral or impartial evidence—with this the ultimate role (and strength) of academic research.^17 18^ However, this fails to recognise that evidence is not something impartial. Rather, research evidence is itself biased by the interests of those involved (eg, the interests of the researchers themselves, but also the interests and biases of the research funder, and of particular journal editors and processes of peer review). This characteristic is particularly important for issues that are highly contested with clashes of actor interests^19^ as are many aspects of achieving food systems transformation—an issue involving some of the wealthiest and most powerful of societal institutions, including MNCs and the fossil fuel industry. Indeed, a number of review studies including work of the nutrition professor Marion Nestle (no relation) have uncovered the bias of studies funded by food and beverage companies in favour of the sponsor’s interests.^20^,^22^

## Conclusion

Support and financial backing of food systems research and development are much needed, particularly in the context of risks to food systems and healthy diets from escalating global challenges including climate change. But achieving such support through engagement and partnerships with industry actors requires caution, if not outright avoidance. Although this new research collaboration with Nestlé is presented as transformative, it appears to draw on the same industry playbook that has run over decades, if not at a quickening pace. Rather than promoting any positive ‘transformation’ in food systems and public health nutrition, this partnership is likely to undermine the transformative change so urgently needed throughout the African continent.

## Data Availability

There are no data in this work.
